# Unmasking the Apex: Multimodality Imaging for the Evaluation of Left Ventricular Apical Obliteration

**DOI:** 10.3390/diagnostics16020184

**Published:** 2026-01-07

**Authors:** Ilaria Dentamaro, Marco Maria Dicorato, Paolo Basile, Maria Cristina Carella, Francesco Mangini, Rita Musci, Roberta Ruggieri, Eduardo Urgesi, Laura Piscitelli, Sergio Dentamaro, Gianluca Pontone, Cinzia Forleo, Marco Matteo Ciccone, Andrea Igoren Guaricci

**Affiliations:** 1 University Cardiology Unit, Interdisciplinary Department of Medicine, Polyclinic University Hospital, University of Bari “Aldo Moro”, 70124 Bari, Italy; paolo.basile@uniba.it (P.B.); m.carella31@phd.uniba.it (M.C.C.); muscir45@gmail.com (R.M.); e.urgesi1@studenti.uniba.it (E.U.); cinzia.forleo@uniba.it (C.F.); marcomatteo.ciccone@uniba.it (M.M.C.); andreaigoren.guaricci@uniba.it (A.I.G.); 2 Cardiology Division, Miulli Hospital, 70021 Acquaviva delle Fonti, Italy; francescomangini.78@libero.it; 3 UOC Cardiologia, Ospedale Di Venere, Asl Bari, 70124 Bari, Italy; roberta.ruggieri1990@gmail.com; 4 Cardiology Unit, A. Perrino Hospital, 72100 Brindisi, Italy; piscitellilaura92@gmail.com; 5 Vascular Surgery, Polyclinic University Hospital, University of Bari “Aldo Moro”, 70124 Bari, Italy; sergio.dentamaro@gmail.com; 6 Centro Cardiologico Monzino IRCCS, 20138 Milan, Italy; gianluca.pontone@cardiologicomonzino.it

**Keywords:** left ventricular apex, apical obliteration, multimodality imaging, cardiac magnetic resonance, apical cardiomyopathies

## Abstract

Left ventricular (LV) apical obliteration represents a convergent imaging phenotype arising from diverse cardiac conditions, including thrombotic, hypertrophic, infiltrative, congenital, and neoplastic diseases. These conditions, despite sharing overlapping morphological features, require profoundly different management strategies. In this context, an accurate characterization of the LV apex is a cornerstone point, and can be performed through various techniques. Advances in multimodality imaging have substantially improved diagnostic precision, allowing clinicians to differentiate true obliteration from mimicking conditions such as hypertrabeculation, apical hypertrophy, or subendocardial fibrosis. This review provides a comprehensive overview of the anatomical variability of the LV apex and its implications for imaging interpretation. We appraise the role of echocardiography, including contrast-enhanced and speckle-tracking studies—alongside cardiac magnetic resonance (CMR), computed tomography (CT), and selective nuclear imaging in the evaluation of apical pathology. For each principal cause of apical obliteration—LV thrombus, apical hypertrophic cardiomyopathy, left ventricular non-compaction, endomyocardial fibrosis, cardiac amyloidosis, and intracardiac tumors—we outline key diagnostic clues, imaging red flags, and distinguishing tissue characteristics. Special emphasis is given to the incremental value of CMR for tissue characterization, thrombus detection, and fibrosis mapping, as well as to the interpretative challenges posed by apical foreshortening, near-field artefacts, and suboptimal acoustic windows. A practical, stepwise imaging framework is proposed to guide clinicians through the differential diagnosis of apical obliteration using an integrated multimodality approach. Future directions include the incorporation of 4D flow, advanced mapping techniques, and artificial intelligence-powered analysis to refine apical phenotyping and identify early disease signatures. Recognizing the full spectrum of apical pathology and its imaging manifestations is essential to prevent misdiagnosis, enable timely therapeutic decisions, and improve risk stratification.

## 1. Introduction

The left ventricular apex (LVA) represents a complex and often underestimated region of the heart whose morphology and function are essential to global systolic performance and to the identification of a wide spectrum of pathologic processes. As emphasized by Cisneros et al., the LV apex is “preferentially affected” by a heterogeneous group of diseases. Its assessment is frequently compromised by acoustic limitations and foreshortening [1]. Because of its anatomical position and tapering myocardial architecture, the LVA may be difficult to fully visualize with conventional echocardiography, leading to under-recognition or misinterpretation of apical abnormalities. The term “apical obliteration” describes a phenotypic endpoint characterized by tissue replacement, thrombus formation, fibrosis or hypertrophy at the LVA, and reflects a confluence of mechanisms rather than a single disease entity. Distinguishing among these entities is imperative, since directly linked to patient’s management. For instance, anticoagulation may be indicated in the setting of an apical thrombus [2], whereas infiltrative diseases such as amyloidosis or endomyocardial fibrosis demand disease-specific therapy. In this context, multimodality imaging plays a central role in the diagnosis and risk stratification of these conditions [3]. The incremental value of combining transthoracic and transesophageal echocardiography with contrast enhancement, together with advanced tissue characterization via cardiac magnetic resonance (CMR) and structural evaluation by computed tomography (CT), has revolutionized our approach to the LVA [3,4,5]. For example, 4-dimensional flow CMR has been shown to detect altered intraventricular hemodynamics particularly at the apex in patients post-MI, offering a potential tool to identify thrombus-prone regions [6]. This narrative review integrates current evidence from peer-reviewed publications and major society recommendations. The figures are provided for illustrative purposes only and do not constitute original research data. Written informed consent for publication was obtained from all patients. The aim of this paper is to provide clinicians and imagers with a practical and clinically oriented framework for the assessment of left ventricular apical obliteration using a multimodality perspective.

## 2. Normal Anatomy and Variability of the Left Ventricular Apex

The LVA represents the distal conical portion of the chamber and constitutes the terminal segment of the three-part subdivision of the ventricle into inlet, apical and outlet components. The myocardial wall at the apex is typically thinner and exhibits fine trabeculations, in contrast to the thicker and more homogeneous basal and mid ventricular walls [7]. On CT, a zone of “physiologic apical thinning” has been documented in healthy subjects, with a mean thickness of approximately 1.2 mm and an extension of about 4.4 mm in the oblique coronal plane [8]. From a morphological standpoint, the LVA exhibits a characteristic conical configuration with a progressively tapering cavity that often confers a “spade-like” appearance on short-axis imaging. The apical myocardium shows a delicate interface between the compact and trabeculated layers, with a variable degree of internal trabeculation among individuals. A relative paucity of papillary muscle structures characterizes the terminal portion of the apex compared with mid-ventricular segments, while the myocardial fibers architecture is specifically arranged to generate apical rotation and basal–apical twist, a mechanism essential for efficient ejection and diastolic suction [9]. These structural features have important implications for imaging and diagnostic interpretation. Because of its thin wall and intricate trabecular pattern, the apex is particularly prone to foreshortening and suboptimal delineation on transthoracic echocardiography, potentially resulting in under-recognition of apical pathology [10]. Furthermore, the natural variability in apical morphology—such as differences in cavity shape, trabecular prominence, and wall thickness—must be appreciated to avoid misdiagnosing normal variants as pathological conditions, for instance, distinguishing physiologic trabeculation in healthy subjects from early left ventricular non-compaction [11]. Finally, the phenomenon of physiological apical thinning, commonly observed even in normal hearts, should not be confused with pathological thinning due to ischemic injury or aneurysm formation [8]. Recognition of this normal variant reinforces the need for imaging modalities with high spatial resolution and adequate tissue contrast [3].

## 3. Imaging Modalities for the LV Apex

### 3.1. Echocardiography

Echocardiography remains the key tool for assessing the LVA, providing a real-time view of wall motion, cavity obliteration, and potential complications. Both transthoracic (TTE) and transesophageal echocardiography (TEE) can visualize apical abnormalities, although image quality may be limited by suboptimal acoustic windows or inherent foreshortening of apical views. Accurate apical imaging requires strict attention to probe angulation and depth, as even minimal foreshortening may obscure or misrepresent apical morphology [12]. TTE is often sufficient to identify apical hypertrophy, fibrosis, or thrombus, especially when harmonic imaging and optimized focus are applied [13,14]. However, in cases of suboptimal endocardial definition, the use of Contrast-enhanced transthoracic echocardiography (contrast TTE) is strongly recommended, in accordance with ASE recommendations [15]. Contrast echocardiography improves the visualization of endocardial borders. It is especially helpful in distinguishing an apical thrombus from pseudo-obliteration caused by near-field artifacts or cavity collapse during systole [16]. Contrast TTE detects LV thrombi with an accuracy almost similar to that of CMR, directing decisions about anticoagulation in ischemic and non-ischemic cardiomyopathies. TEE may assist when TTE visualization is limited by different causes, such as obesity or mechanical ventilation. Multiplane and deep gastric views occasionally offer even additional diagnostic information [17]. Despite its central role, echocardiography has inherent limitations in evaluating the apex. The thin-walled apical myocardium and near-field position are prone to dropout and artifact [12], and the technique remains dependent on operator expertise, acoustic windows, and guideline-based restrictions surrounding contrast agent use, which may affect diagnostic consistency. Tissue characterization is limited, complicating the differentiation among thrombus, hypertrophied myocardium, and fibrosis. These limitations underscore the value of integrating echocardiographic findings with CMR or CT for comprehensive assessment [18].

### 3.2. Cardiac Magnetic Resonance

CMR has emerged as the gold standard for detailed evaluation of the LVA due to its high spatial resolution, independence from acoustic window constraints, and possibility of tissue characterization [19]. It allows precise assessment of apical shape, wall motion, and myocardial composition, supporting differentiation between true apical obliteration and conditions such as thrombus, apical hypertrophic cardiomyopathy (ApHCM), or infiltrative disease [20,21]. Cine sequences provide high-resolution visualization of chamber morphology throughout the cardiac cycle, enabling accurate identification of obliteration patterns and regional wall motion abnormalities [22]. The “spade-like” configuration of the apical cavity in ApHCM and the systolic collapse associated with thrombus can be clearly evidenced [11]. Late gadolinium enhancement (LGE) sequences are fundamental for tissue characterization, distinguishing non-enhancing thrombus from fibrotic or hypertrophied myocardium, which exhibit variable enhancement depending on etiology. LGE is particularly valuable in differentiating ischemic from non-ischemic causes of apical obliteration and in detecting subtle endomyocardial fibrosis in hypereosinophilic syndromes [20]. Parametric mapping techniques, including T1 and T2 mapping, quantify tissue composition; T2 mapping identifies edema and inflammatory or infiltrative processes, whereas native T1 mapping detects diffuse fibrosis or infiltration. These methods increase diagnostic precision when standard LGE findings are inconclusive, such as in small thrombi or early amyloidosis. However, mapping is influenced by vendor-specific implementations and inter-scanner variability, limiting standardization across centers [23]. CMR also offers three-dimensional and multiplanar evaluation of the apex, enabling accurate measurement of cavity size, wall thickness, and the degree of obliteration. This comprehensive evaluation supports clinical decision-making, including anticoagulation for thrombus, targeted therapy for infiltrative cardiomyopathies, and surgical planning in obstructive hypertrophic phenotypes [24].

### 3.3. Computed Tomography

When echocardiography or CMR are inconclusive or contraindicated, multidetector CTprovides excellent spatial resolution and is especially useful for anatomical characterization of the LV apex [25,26]. CT delineates myocardial thickness, trabeculations, calcifications, and apical contour with high precision, although it lacks the intrinsic tissue characterization of CMR [27]. Low-attenuation, non-enhancing apical filling defects may indicate thrombus, and CT is particularly valuable in patients with CMR-incompatible devices or renal dysfunction precluding gadolinium use, although CMR remains the gold standard for thrombus detection [28]. High-resolution CT can also distinguish thrombus from adjacent structures such as prominent trabeculations or papillary muscle remnants [29]. CT is crucial in distinguishing true versus false aneurysms and in assessing myocardial wall integrity, providing information essential for surgical planning [30]. It is highly effective in detecting apical calcifications—whether post-infarction or associated with endomyocardial fibrosis—and can differentiate chronic fibrotic obliteration from active inflammatory or thrombotic processes through identification of dense “eggshell” calcific plates [31]. CT is even more important when apical obliteration coexists with congenital variants or coronary anomalies, offering detailed multiplanar and 3D reconstructions [24,32]. It can also help differentiate ApHCM from other forms of apical thickening by providing precise measurements of wall thickness and cavity geometry [33]. Since CT requires ionizing radiation, a comprehensive evaluation of patient’s age, renal and allergic risks is requested. Despite not considered a first-line imaging modality for apical evaluation, the selective use of CT can provide important complementary information in this setting.

## 4. Differential Diagnosis of LV Apical Obliteration

### 4.1. Apical Thrombosis

**Figure 1:**
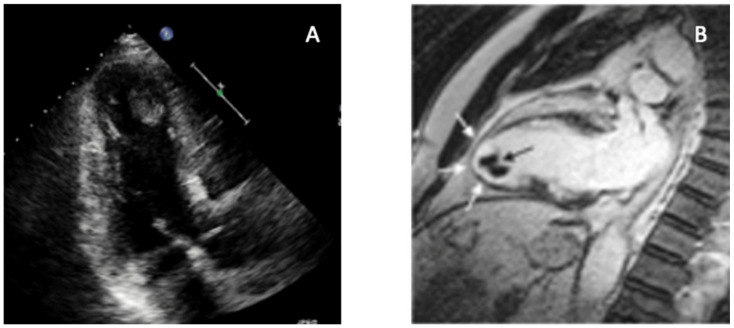
Multimodality imaging of left ventricular apical thrombus. Key teaching point: Combining contrast-poor echocardiography with CMR allows definitive differentiation between apical thrombus and other causes of apical obliteration such as ApHCM, EMF, or LVNC. (**A**) Transthoracic echocardiography (apical four-chamber view) demonstrates a well-defined, hyperechoic mass adherent to the LV apex, associated with severe regional wall-motion abnormality. (**B**) CMR cine imaging confirms an apical thrombus (black arrow), visualized as a non-enhancing, low-signal mass sharply distinct from the surrounding dyskinetic myocardium (white arrows). Multimodality evaluation enables confident identification of apical LVT and prevents misdiagnosis as hypertrophic, infiltrative, or fibrotic apical disease, directly guiding anticoagulation and clinical management. Original image from our institution (single patient).

The most frequent cause of apical obliteration is apical thrombosis, a type of left ventricular thrombosis (LVT) marked by the formation of a blood clot inside the apical cavity. It has significantly higher risks of systemic embolism, stroke, cardiovascular events, and death. It is usually caused by blood stasis in severe ventricular systolic dysfunction of both ischemic and non-ischemic etiologies [34]. Up to 15% of patients with STEMI, 25% of patients with anterior STEMI, and 2–36% of patients with non-ischemic cardiomyopathies experience LVT [34]. Large anterior infarction, severe left ventricular dysfunction, microvascular obstruction (MVO), and delayed reperfusion are important predictors of this occurrence [35]. TTE represents the first-line screening method because of its accessibility and quick acquisition, even though sensitivity remains low [36]. Apical visualization may be limited by near-field clutter, foreshortening, and suboptimal acoustic windows. Standard diagnostic criteria include a mass with distinct echogenicity, well-defined margins, visualization in at least two orthogonal views, and contiguity with an akinetic or dyskinetic segment [35]. Additional echocardiographic markers of stasis, such as reduced apical longitudinal strain or a low E-wave propagation index (EPI < 1), have shown good sensitivity but limited specificity for thrombus detection [35]. Contrast TTE significantly improves endocardial border delineation and LV cavity opacification, frequently doubling thrombus detection compared with non-contrast TTE, particularly for mural or laminated thrombi [37,38]. CMR is the gold standard for apical LVT detection due to superior spatial resolution and unique tissue characterization potential [39]. Thrombus appears as a hypointense, non-enhancing mass on LGE imaging; long-inversion-time sequences (~600 ms) markedly improve conspicuity and outperform cine imaging [35]. These findings are consistent with multicenter data and meta-analytic evidence demonstrating the superior sensitivity of CMR over TTE for LVT detection [35,40]. CMR is especially useful for detecting small, mural, or laminated thrombi frequently missed by echocardiography [40]. LGE burden strongly correlates with thrombus formation, with ischemic cardiomyopathy demonstrating a fivefold increased prevalence compared with non-ischemic disease [41,42]. CMR also differentiates thrombus from MVO, the latter being intramyocardial and surrounded by hyperenhancement [7]. Emerging tools—including targeted contrast agents and 4D flow MRI—may further refine thrombus characterization and risk prediction [9,43]. Cardiac CT angiography (CCTA) offers high spatial resolution and rapid acquisition when CMR is contraindicated [29]. Its utility is balanced by radiation exposure and the need for iodinated contrast. Thrombus is distinguished from apical hypertrophic cardiomyopathy, endomyocardial fibrosis (EMF), and tumors by its avascular and non-enhancing profile. Heterogeneous enhancement suggests tumor, whereas subendocardial fibrosis or calcification are more typical of EMF. Since prompt anticoagulation for at least three months lowers embolic risk and may affect long-term outcomes, early and accurate identification of LVT is crucial. Radiation exposure and need for iodinated contrast represent important limitations. Moreover, comparative data with CMR are still limited [35]. The multimodality appearance of apical LVT, including the distinction between non-enhancing thrombus and surrounding dyskinetic myocardium, is shown in Figure 1.

### 4.2. Apical Hypertrophic Cardiomyopathy

**Figure 2:**
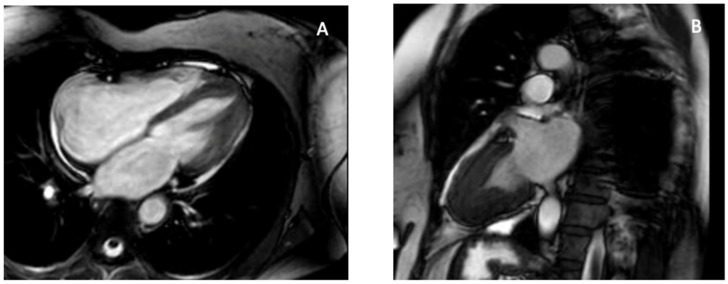
CMR features of apical hypertrophic cardiomyopathy. CMR provides definitive characterization of ApHCM by demonstrating apical wall thickening and the classic “spade-shaped” configuration, allowing differentiation from thrombus, LVNC, and EMF. (**A**) Four-chamber SSFP cine image at end-diastole shows marked hypertrophy confined to the left-ventricular apex with apical displacement of the papillary muscle. (**B**) Two-chamber SSFP cine view demonstrates the characteristic “spade-shaped” LV cavity, reflecting disproportionate apical wall thickening. These imaging hallmarks distinguish ApHCM from other causes of apical obliteration or pseudo-obliteration, particularly thrombus (avascular mass), LVNC (deep perfused recesses), and EMF (subendocardial fibrosis and true apical cavity loss). Original images from our institution (single patient).

Apical hypertrophic cardiomyopathy (ApHCM), or Yamaguchi syndrome, was first reported in Japan in 1976 [44], and is defined by unexplained left ventricular hypertrophy predominantly involving the apex, with an end-diastolic apical wall thickness ≥15 mm [45]. Sarcomeric mutations in MYH7 and MYBPC3 are identified less frequently compared with other HCM subtypes and do not significantly affect clinical outcomes [46]. Clinical presentation ranges from asymptomatic to chest pain, dyspnea, or syncope, and myocardial ischemia may result from apical hypertrophy, microvascular dysfunction, and increased intracavitary pressures. Apical aneurysms develop in up to 13% of patients and are associated with arrhythmic and thromboembolic risk [47,48]. TTE is the first-line modality for diagnosis and follow-up [11]. ApHCM typically lacks septal hypertrophy or systolic anterior motion—features that distinguish it from classic HCM—and is generally not associated with LV outflow tract obstruction. Instead, hypertrophy is confined to the mid-to-apical LV segments, producing the characteristic “ace of spades” configuration. Subtypes include pure ApHCM (hypertrophy below papillary muscles), mixed ApHCM (involving papillary level), and relative ApHCM (milder thickening not meeting the ≥15 mm threshold). Apical systolic cavity obliteration, quantified by an end-systolic obliteration ratio >0.5, correlates with adverse prognosis [11]. Severe cases may develop paradoxical flow reversal and apical aneurysm formation. Functional assessment with echocardiography shows impaired diastolic filling and reduced apical strain, creating an “inverse-amyloid” deformation pattern; disease progression leads to diminished apical twist and lower global rotational mechanics [49,50,51]. 3D-TTE enables more accurate volumetric and mass assessment and may detect subtle asymmetric apical hypertrophy using parameters such as the Mass Dispersion Index [52,53]. TEE has limited utility due to poor apical visualization and infrequent mitral valve abnormalities in pure ApHCM [54]. CMR is the preferred modality because of superior apical resolution. Cine imaging detects hypertrophy and aneurysms—missed by TTE in up to 40% of cases [23]. LGE burden >15% is associated with increased arrhythmic risk [55] while LGE and T1/T2 mapping characterize fibrosis and help distinguish ApHCM from phenocopies, including Fabry disease (low T1) [56] and cardiac amyloidosis (high T1 and ECV [57]. The characteristic apical hypertrophy and spade-shaped systolic cavity configuration are illustrated in Figure 2. Cardiac CT provides high-resolution anatomic imaging, quantifies iodine-based delayed enhancement, and evaluates coronary arteries and myocardial bridges and 4D-CT can delineate obstruction and small apical aneurysms [33]. Nuclear techniques such as SPECT and Positron emission tomography (PET) assist in distinguishing ApHCM from coronary artery disease or other LV hypertrophy etiologies, with ApHCM demonstrating the distinctive “Solar Polar” pattern on thallium-201 maps and unique apical perfusion defects on PET [33,58]. ApHCM can mimic other apical pathologies. Unlike thrombus, hypertrophied myocardium shows contractility and LGE rather than a non-enhancing mass. Accurate identification of ApHCM is essential because apical aneurysms, arrhythmias, ischemia, and progressive diastolic dysfunction may occur despite preserved global systolic function. Imaging-derived markers—particularly apical LGE burden, aneurysm size, and strain parameters—guide risk stratification, surveillance, and therapeutic decision-making.

### 4.3. Left Ventricular Non-Compaction

**Figure 3:**
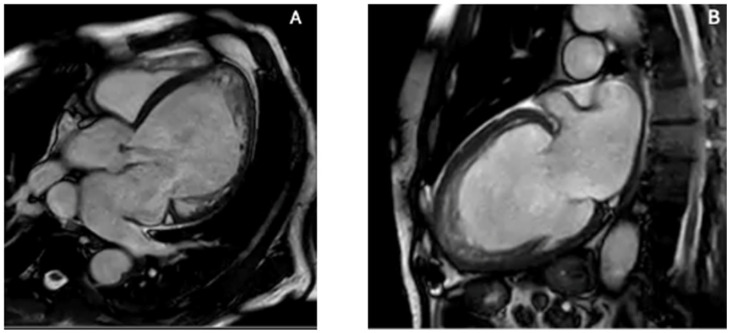
CMR features of left ventricular non-compaction. CMR provides superior delineation of trabecular anatomy, enabling confident distinction between true LVNC and other causes of apparent apical obliteration such as thrombus, ApHCM, and EMF. (**A**) Four-chamber SSFP cine image demonstrates a markedly trabeculated apex with deep intertrabecular recesses and a thin compacted myocardial layer. (**B**) Two-chamber SSFP cine view confirms prominent apical trabeculation and recess communication with the ventricular cavity, fulfilling morphological criteria for LVNC. The combination of excessive trabeculation, deep perfused recesses, and reduced compacted layer thickness differentiates LVNC from ApHCM (which shows compacted hypertrophy), EMF (subendocardial fibrosis with true apical cavity loss), and apical thrombus (avascular mass without recess perfusion). Original images from our institution (single patient).

Left ventricular non-compaction (LVNC) is one of the most challenging differential diagnoses when evaluating apparent apical obliteration [59,60,61]. As emphasized in the 2023 ESC Cardiomyopathy Guidelines [62], LVNC is now considered a morphological trait rather than a distinct cardiomyopathy, since excessive trabeculation may occur physiologically (e.g., pregnancy, athletic remodeling) or across multiple pathological phenotypes, including dilated, hypertrophic, and arrhythmogenic cardiomyopathies [59,63]. Although traditionally attributed to incomplete embryogenic compaction between weeks 5–8 [59,64], contemporary evidence demonstrates that hypertrabeculation may also be acquired or adaptive and may regress when the inciting stimulus resolves [63]. Genetic studies have shown how pathogenic variants in MYH7, MYBPC3, TNNT2, and ACTC1—classically associated with HCM or DCM—are common in LVNC, with marked familial heterogeneity, incomplete penetrance, and variable expressivity [59,65,66]. TTE is the first-line modality for identifying LVNC, but diagnostic criteria vary widely. Early definitions include the Chin ratio (X/Y ≤ 0.5) [67], Jenni’s end-systolic NC/C ratio >2 with color Doppler evidence of perfused recesses [64] and Stöllberger’s requirement of ≥3 prominent trabeculations with intertrabecular spaces perfused from the LV cavity [68]. However, all criteria show modest reproducibility and tend to overdiagnose LVNC, especially in patients with ventricular dilation or hypertrophy [59]. In addition, apical foreshortening, poor acoustic windows, and anatomical complexity may hinder differentiation between true non-compaction and obliteration from thrombus, fibrosis, or hypertrophy [69,70]. Contrast TTE improves endocardial border delineation and allows visualization of recess perfusion: microbubble opacification within deep trabecular spaces supports LVNC, whereas absent perfusion favors thrombus or fibrotic obliteration [71]. Functional techniques, including strain imaging and tissue Doppler, demonstrate abnormal apical rotation or paradoxical twist mechanics, reflecting disrupted fiber architecture and supporting the diagnosis [71]. CMR has transformed LVNC evaluation. Petersen’s diastolic NC/C ratio ≥2.3 yields high sensitivity and specificity [72]. These CMR criteria have been validated in large cohorts and are now integrated into contemporary diagnostic algorithms for LVNC [62,72,73]. while Jacquier’s trabeculated mass >20% of LV mass and Grothoff’s threshold of ≥25% non-compacted mass—with basal involvement—predict pathological LVNC and adverse outcomes [65,73]. Tissue characterization further refines differential diagnosis: isolated LVNC typically exhibits minimal or absent fibrosis, whereas ApHCM demonstrates localized thickening with patchy LGE [74]. CMR feature-tracking strain may reveal reduced apical rotation or mechanical dispersion [63]. CMR-detected hallmarks of LVNC, including excessive trabeculation, deep recesses, and thin compacted layer are shown in Figure 3. Cardiac CT provides high-resolution delineation of apical contours and may clarify recess morphology, compacted/non-compacted transitions, or confounding variants when echocardiography and CMR are inconclusive [74]. Correct identification of LVNC in the context of apical obliteration is essential due to its association with arrhythmias, thromboembolism, and systolic dysfunction [75,76]. Conditions mimicking LVNC include ApHCM, apical LVT, and restrictive or hypertensive cardiomyopathies. In ApHCM, wall thickness exceeds 15 mm with preserved compacted myocardium and characteristic LGE [60]. Thrombus appears as a non-enhancing mass on LGE-CMR or contrast TTE [77]. Restrictive and hypertensive cardiomyopathies may cause apparent apical obliteration but lack deep perfused recesses [65]. Some patients show overlapping LVNC–HCM–ApHCM features, supporting a morpho-functional continuum [66].

### 4.4. Endomyocardial Fibrosis/Hypereosinophilic Syndrome

**Figure 4:**
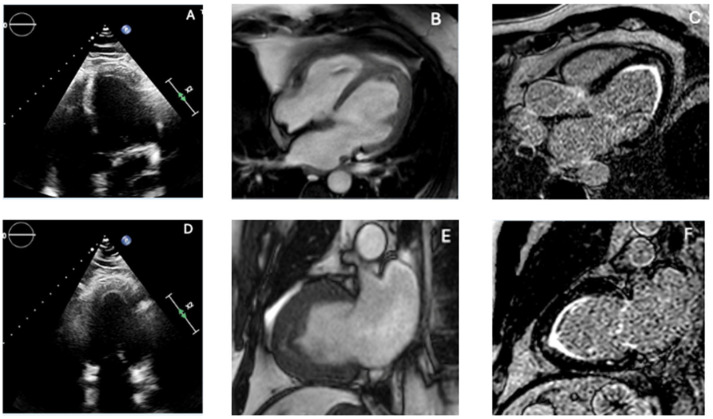
Multimodality imaging features of endomyocardial fibrosis. Combining echocardiography and CMR is essential to differentiate true apical obliteration from thrombus, hypertrophic variants, and infiltrative cardiomyopathies, as EMF shows a characteristic pattern of subendocardial fibrosis with or without overlying thrombus. (**A**,**D**) Transthoracic echocardiography reveals marked apical obliteration with increased endocardial echogenicity, reduced cavity size, and features consistent with restrictive physiology. (**B**,**E**) CMR cine images demonstrate apical thickening and impaired ventricular compliance, with reduction or loss of the apical cavity. (**C**,**F**) Late gadolinium enhancement (LGE) sequences show the typical subendocardial fibrotic pattern of EMF, frequently accompanied by an overlying apical thrombus, producing the characteristic “double V” sign. The combination of subendocardial LGE, apical cavity loss, and associated thrombus strongly supports EMF and differentiates it from ApHCM (compact hypertrophy with patchy LGE), LVNC (deep perfused recesses), and apical LVT (avascular mass without subendocardial fibrosis). Original images from our institution (single patient).

EMF represents the most frequent cause of restrictive cardiomyopathy in tropical areas [78]. Although its exact cause is unknown, hypereosinophilia is frequently linked to myocardial damage, which can lead to thrombus formation, dense endomyocardial fibrosis, apical obliteration, and atrioventricular valve dysfunction [78]. The diverse clinical profile and frequent overlap with other systemic or cardiac diseases can be attributed to the coexistence of the classical necrotic, thrombotic, and fibrotic phases at presentation [79]. Imaging is essential for diagnosis, staging, and follow-up because of this variability. TTE is the first-line modality. Typical findings include bright, thickened endomyocardium; apical cavity obliteration, frequently associated with mural thrombi, biatrial enlargement, and a restrictive filling pattern [80]. Thrombi in EMF may appear low-echogenic with central brightness and can occur despite preserved regional wall motion—an observation that helps differentiate EMF from post-ischemic thrombi [80]. Extension of the thrombotic–fibrotic process into the subvalvular apparatus may tether chordae and papillary muscles, generating eccentric mitral or tricuspid regurgitation in advanced disease [81]. Doppler parameters typically show severe diastolic dysfunction with E/A > 2.5, short E wave deceleration time (<150 ms), reduced e′ velocities, elevated E/e′ ratio, and markedly increased left atrial volume index (>50 mL/m^2^) [82]. Pericardial effusion may be present in 10–32% of cases, particularly when myocarditis predominates [83]. TEE and 3D-TTE offer incremental information when transthoracic images are suboptimal, but are limited by reduced view of apical field [84]. Contrast TTE is a valuable tool for distinguishing thrombus from trabeculation or fibrotic tissue. Actually, absence of microbubble penetration supports an avascular etiology, such as thrombus or dense fibrosis, whereas microbubble entry into intertrabecular recesses suggests alternative diagnoses such as LVNC [85]. Speckle-tracking echocardiography may reveal reduced global longitudinal strain (GLS) as an early marker of subclinical dysfunction [86]. CMR is the reference technique for comprehensive evaluation and should include cine SSFP imaging, T1/T2-weighted sequences, perfusion imaging, and contrast-enhanced LGE sequences, with long-inversion-time acquisitions when thrombus is suspected [87]. CMR classically identifies subendocardial thickening with delayed enhancement, apical obliteration often with overlying thrombus—producing the characteristic “double V” sign—and secondary atrial enlargement with atrioventricular regurgitation [20,88]. The main imaging features of EMF, including the subendocardial “double V” LGE, are shown in Figure 4. The typical scar pattern is non-ischemic, reflecting inflammation, infiltration, or replacement fibrosis [89]. Multiple fibrosis patterns (types 1–5) correlate with symptom severity and surgical candidacy [83]. CMR also distinguishes thrombus from fibrotic tissue and evaluates right ventricular involvement, both essential for prognosis and surgical planning. EMF must be differentiated from ApHCM, LVNC, and apical LVT. ApHCM retains compacted myocardium with hypertrophy ≥15 mm and characteristic LGE patterns. LVNC demonstrates deep perfused recesses absent in EMF. On LGE, apical LVT exhibits non-enhancement without subendocardial thickening. Since the degree of fibrosis, thrombus burden, and valvular involvement all affect prognosis, accurate EMF identification is essential [83]. Anticoagulation, arrhythmia monitoring, and consideration of endocardiectomy or valve repair in advanced stages are possible by an early and accurate diagnosis.

### 4.5. Cardiac Amyloidosis and Atypical Apical Involvement

**Figure 5:**
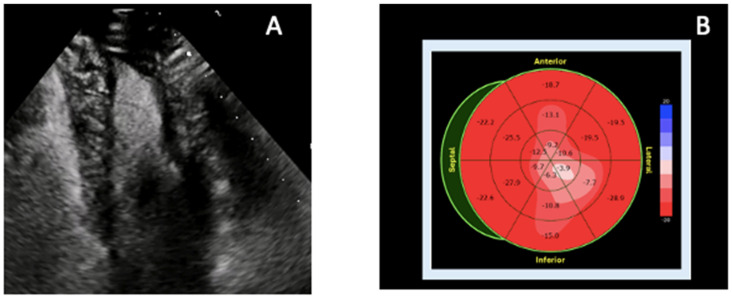
Echocardiographic features of atypical apical involvement in cardiac amyloidosis. In cardiac amyloidosis, apical thickening may mimic ApHCM or focal fibrosis, but multimodality echocardiographic assessment—particularly contrast TTE combined with strain analysis—reveals characteristic patterns that help differentiate infiltrative disease from other apical pathologies. (**A**) Contrast-enhanced TTE (apical two-chamber view) demonstrates improved endocardial border delineation and increased biventricular wall thickness with a granular sparkling myocardial texture, supportive of atypical apical amyloid infiltration. (**B**) Speckle-tracking-derived bull’s-eye plot shows disproportionately reduced apical longitudinal strain (“reverse apical sparing”), a deformation pattern associated with apical AL amyloidosis and distinct from the classic RELAPS pattern of ATTR. The combination of granular apical texture, contrast-enhanced visualization, and apex-predominant strain reduction differentiates amyloidosis from ApHCM (where apical strain is typically preserved or only mildly reduced), ischemic scarring (territorial pattern), and LVNC (deep perfused recesses rather than homogeneous thickening). Original images from our institution (single patient).

Cardiac amyloidosis (CA) results from myocardial deposition of misfolded fibrillary proteins demonstrating Congo Red affinity and yellow–green birefringence under polarized light [90,91]. Among the over 30 amyloidogenic proteins, immunoglobulin light chains (AL) and transthyretin (TTR)—either hereditary (vATTR) or wild-type (wtATTR)—represent the most frequent and significant for cardiac involvement [92]. Penetrance varies by TTR mutation, wtATTR predominantly affects the heart, and nearly 70% of AL patients develop cardiac disease [90]. Amyloid infiltration leads to progressive myocardial thickening, diastolic dysfunction, and heart failure, often mimicking hypertensive heart disease or hypertrophic cardiomyopathy in early stages [57], and is now explicitly recognized among key etiologies of HFpEF in contemporary heart failure guidelines [93]. Echocardiography is the first-line tool that raise suspicion of CA. It typically shows increased wall thickness, low-voltage ECG–echo mismatch, biatrial enlargement, valvular thickening, and elevated filling pressures [57,94]. GLS, assessed through speckle-tracking, demonstrates relative apical sparing (RELAPS), with less impaired apical strain compared with basal segments [95,96], likely related to differential amyloid deposition or remodeling gradients [97]. An apical-to-mid-basal LS ratio >1 provides high sensitivity (93%) and specificity (82%) for distinguishing CA from other LV hypertrophy etiologies [90]. Careful avoidance of apical foreshortening is essential, as this may falsely exaggerate apical strain [98]. ATTR is typically associated with concentric hypertrophy with RELAPS. On the other hand, atypical apical variants are increasingly recognized in AL amyloidosis. Cytotoxic light chains may promote microvascular dysfunction, necrosis, and replacement fibrosis, producing heterogeneous strain patterns—including true apical involvement and “reverse apical sparing” [90]. The appearance of atypical apical amyloid infiltration on contrast TTE and the corresponding strain abnormalities are illustrated in Figure 5. Apical thickening, fibrosis, and intracavitary thrombus formation may occur, contributing to low-flow states and embolic risk even in sinus rhythm [99]. These thrombi may be challenging to detect on standard TTE, necessitating Contrast TTE or CMR. Echocardiographic features suggestive of apical involvement include granular apical thickening, apparent apical pseudo-obliteration, loss of RELAPS, and difficulty in distinguishing thrombus from infiltrated myocardium [100]. CMR provides critical tissue characterization, and LGE, native T1 mapping, and extracellular volume quantification reveal amyloid deposition, fibrosis, and apical involvement [101]. The prognostic and diagnostic value of these mapping parameters has been confirmed in multicenter cohorts and meta-analyses [90,102]. CMR also differentiates true apical obliteration—typical of EMF—from pseudo-obliteration due to massive amyloid infiltration [102,103,104]. Feature-tracking CMR confirms longitudinal strain impairment with relative apical sparing, paralleling echocardiographic findings [105]. Integration of echocardiography, strain imaging, and CMR is crucial because of the aggressive course of AL amyloidosis and the increased risk of thrombotic and embolic complications.

### 4.6. Intracardiac Tumors

Intracardiac tumors are an uncommon but clinically important cause of LV apical obliteration or pseudo-obliteration [106,107]. Primary cardiac neoplasms are exceedingly rare, with an autopsy incidence of 0.056%, whereas metastatic involvement is far more common (≈1.23%) [108]. Despite their rarity, apical tumors are diagnostically relevant because they can closely mimic far more prevalent apical pathologies such as thrombi, ApHCM, EMF, LV non-compaction, or focal infiltrative disease including amyloidosis [106]. Small, infiltrative, or trabeculae-embedded apical masses may remain occult on routine echocardiography and should be suspected whenever a non-thrombotic cause of apical obliteration is considered [109]. Apical obliteration from neoplastic disease may arise through cavity-occupying masses, wall-infiltrating lesions, chronic compression with remodeling, or pseudo-obliteration due to limited contrast between tumor tissue and adjacent trabeculations. These patterns occur across both benign and malignant entities. Metastatic disease is much more frequent—20–40 times more common than primary cardiac tumors [110]. Common sources include melanoma, lung and breast carcinoma, esophageal cancer, and hematologic malignancies [107]. Case reports highlight metastatic melanoma misdiagnosed as apical hypertrophy until clarified by PET imaging [111], renal cell carcinoma metastasis to the LV without vena cava involvement [112], and lung carcinoma infiltrating the apex presenting as acute coronary syndromes [113]. Primary malignant lesions such as angiosarcomas may infiltrate the LV free wall or apex, altering geometry and restrictive physiology [114]. Benign tumors including fibromas, rhabdomyomas, myxomas, and papillary fibroelastomas may also affect the apex or extend into it [115,116,117]. TTE remains the first-line modality for assessing size, mobility, echogenicity, and relationships with adjacent structures [118], but it often lacks specificity for small or infiltrative apical lesions. TEE or Contrast TTE may reveal vascularity—an important discriminator, as thrombi are avascular [119]. CMR is pivotal for tissue characterization. Neoplastic masses typically show intermediate–high T2 signal, variable T1, and early or heterogeneous LGE due to vascularity and necrosis; in contrast, apical thrombi show low signal intensity and no contrast uptake [120]. CMR also identifies infiltrative patterns, wall thickening, pericardial involvement, and extension into the right ventricle—findings that redirect the differential away from ApHCM or EMF. FDG-PET is a valuable tool in distinguishing metabolically active tumors from chronic thrombi or fibrotic obliteration, while CT defines calcification, fat content, and extracardiac extension [121]. Tumors should be considered when imaging findings are discordant with ApHCM or EMF, when enhancement patterns are atypical for fibrosis, when apical masses persist despite anticoagulation, or when systemic features (constitutional symptoms, unexplained effusion, multi-organ involvement) are present. Correct identification of apical tumors has major therapeutic implications. Neoplastic lesions may require surgical excision, systemic therapy, or oncologic evaluation, whereas ApHCM and EMF demand targeted cardiomyopathy management and thrombi require anticoagulation [109]. Awareness of tumor-related apical mimicry is essential to avoid misdiagnosis and ensure appropriate treatment.

## 5. A Practical Multimodality Imaging Approach

**Figure 6:**
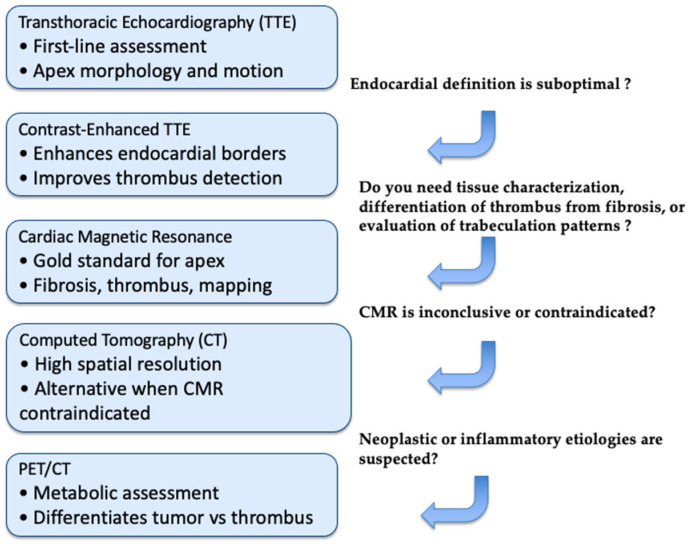
Diagnostic Workflow for LV Apical Obliteration. This stepwise imaging algorithm outlines the optimal use of echocardiography, CMR, CT, and PET/CT in the evaluation of suspected LV apical obliteration. Initial assessment relies on standard TTE, with contrast enhancement when endocardial definition is limited. CMR is preferred for tissue characterization, thrombus–fibrosis differentiation, and evaluation of trabeculation patterns. CT is indicated when CMR is inconclusive or contraindicated, providing high-resolution anatomical detail. PET/CT is reserved for cases in which neoplastic or inflammatory etiologies are suspected.

A structured multimodality imaging strategy is essential for the evaluation of left-ventricular apical obliteration and apical mass-like conditions. These entities share overlapping morphological features that range from hypertrophic phenotypes to thrombotic, infiltrative, or neoplastic processes. Thus, a single technique is not sufficient to reach an accurate diagnosis. Instead, a stepwise, problem-oriented approach allows clinicians to leverage the strengths of each modality and progressively narrow the differential diagnosis. The first-line technique is TTE, which offers quick evaluation of wall thickness, and apical morphology and Doppler hemodynamics [12]. However, extra testing is frequently necessary for apical foreshortening, inadequate acoustic windows, and limited tissue characterization. By helping to distinguish avascular thrombus from solid masses or hypertrophic myocardium, Contrast TTE enhances endocardial definition [16]. Strain imaging further increases the diagnostic accuracy, in particular when characteristic profiles are present, such as apical sparing in CA or marked apical impairment in EMF and apical LVT. When preliminary imaging tools are not conclusive, CMR represents a mandatory second step approach, and is often considered the more accurate and complete non-invasive technique. Indeed, CMR can accurately differentiate fibrosis from infiltration, HCM from restrictive pathologies, and thrombus from tumors thanks to its high spatial resolution, late gadolinium enhancement patterns, and quantitative mapping [23]. In specific contexts, CT or FDG-PET can add diagnostic information, especially when calcification, extracardiac extension, or metabolic activity needs to be evaluated. Lastly, endomyocardial biopsy remains the gold standard when imaging raises suspicion for infiltrative disease, EMF, or neoplasm and a definitive diagnosis is supposed to change patient’s treatment [33]. A useful, step-by-step framework for supporting consistent evaluation across diverse clinical presentations is provided by Table 1. To standardize the diagnostic approach and minimize overlap among apical pathologies, we propose a stepwise multimodality imaging algorithm (Figure 6). Initial assessment relies on transthoracic echocardiography, followed by contrast-enhanced studies when endocardial definition is suboptimal. CMR is the preferred second-line modality for comprehensive tissue characterization, differentiation of thrombus from fibrosis, and evaluation of trabeculation patterns. CT is reserved for cases where CMR is inconclusive or contraindicated, and PET imaging is selectively used when neoplastic or inflammatory aetiologies are suspected. This structured pathway aims to improve the diagnostic accuracy in this setting, guiding targeted management.

## 6. Conclusions and Future Prospective

LVA obliteration represents a final common morphological pathway shared across a broad spectrum of pathological substrates—including thrombotic, infiltrative, hypertrophic and fibrotic diseases. The evidence reviewed in this manuscript reinforces the notion that multimodality imaging is indispensable for distinguishing among these entities, guiding tailored therapeutic strategies, and recognizing patients with greater risk of adverse clinical outcomes. Echocardiography remains the cornerstone tool for a first-line assessment, particularly through enhanced border delineation with contrast agents and the incremental diagnostic value of deformation imaging. CMR has emerged as the reference modality for tissue characterization, enabling precise differentiation between thrombus and pathological tissue, detailed mapping of subendocardial fibrosis in EMF, and identification of LGE or mapping abnormalities across the heterogeneous phenotypes of CA. CT and nuclear imaging, although more selectively applied, provide relevant complementary information in specific clinical scenarios, particularly for calcific apices, intracardiac masses, or infiltrative disorders [28]. In the future, technological developments have the potential to empower the diagnostic accuracy of LVA pathology. The reproducibility and sensitivity of apex-specific parameters will probably be improved by artificial intelligence (AI)-driven image analysis, which will get around issues with foreshortening and less-than-ideal acoustic Windows [122,123,124,125]. Particularly in ischemic cardiomyopathy and apical aneurysms, 4D flow CMR has demonstrated promising results for measuring local hemodynamics and thrombotic risk [126]. Even in environments with limited resources, a more comprehensive integration of quantitative T1/ECV mapping may enhance the early detection of infiltrative diseases before irreversible apical remodeling occurs [23]. Hybrid approaches combining PET and CMR may further refine assessment in amyloidosis, particularly in atypical patterns where apical involvement or sparing complicate diagnosis [127]. Future research should also address unresolved controversies, including the true prevalence of morphological overlaps between ApHCM, LVNC, and infiltrative phenotypes. Although emerging AI-based tools show promise for automated detection of apical abnormalities and improved tissue characterization, several challenges still limit their clinical integration. These include the need for large, well-annotated datasets, variability in acquisition protocols and vendor-specific sequences, and limited inter-center generalizability of trained models. Continued standardization and multicenter validation will be essential before AI can reliably complement multimodality imaging workflows. Large, prospective multicenter studies are also required to define standardized diagnostic algorithms and identify outcome-relevant imaging markers, with particular attention to thrombotic complications, arrhythmic risk, and progression toward restrictive physiology. Ultimately, a structured multimodality workflow—integrating clinical context, advanced imaging, and genetic or biomarker data—represents the most reliable strategy to “unmask” the LV apex and guide precision medicine in this complex area of cardiology.

## Figures and Tables

**Table 1:** Multimodality imaging features of left-ventricular apical obliteration condition.

Condition	TTE (2D/Contrast)	Strain Imaging	CMR (Cine, LGE, Mapping)	CT/PET (Selected Use)
**Apical HCM**	Apical wall thickness ≥15 mm; systolic cavity obliteration; apical aneurysm possible	Preserved or mildly reduced apical strain; increased basal strain	Focal apical hypertrophy; patchy LGE; normal/mildly ↑ T1	CT useful if poor echo window
**Endomyocardial Fibrosis**	Apical obliteration; restrictive filling; mural thrombus common; thickened endocardium	Marked apical strain reduction; basal-apical gradient reversed	Subendocardial LGE (“V-sign”); apical fibrosis; ↑ T1/ECV	CT shows calcification of apex/endocardium
**Cardiac Amyloidosis**	Concentric hypertrophy; sparkling myocardium; biatrial enlargement; possible apical sparing	Typical apical sparing of LS; high apical/basal ratio	Global/subendocardial LGE; nulling difficulties; ↑ T1/ECV	Bone scintigraphy for ATTR; PET rarely complementary
**Apical Thrombus**	Avascular mass; improved visibility with contrast	Markedly reduced or absent apical strain	No LGE; very low T1/T2; distinct borders	CT helps assess calcified thrombi
**Intracardiac Tumors**	Solid, mobile or sessile mass; variable enhancement with CE	Focal segmental strain reduction	Heterogeneous enhancement; infiltration patterns; variable T1/T2	PET for metabolic activity; CT for fat/calcification
**LV Noncompaction**	Prominent trabeculations; deep recesses; ratio >2:1	Regional strain heterogeneity	Hypertrabeculated apex; non-ischemic LGE possible	CT helps confirm anatomy

Key echocardiographic, strain, CMR, and CT/PET findings are summarized for ApHCM, endomyocardial fibrosis, cardiac amyloidosis, apical thrombus, intracardiac tumors, and LV noncompaction, highlighting characteristic morphologic, functional, and tissue signatures.

## Data Availability

The original contributions presented in this study are included in the article. Further inquiries can be directed to the corresponding authors.

## Abbreviations

4D Flow	Four-dimensional flow magnetic resonance imaging
AL	Immunoglobulin light-chain amyloidosis
ApHCM	Apical hypertrophic cardiomyopathy
ATTR	Transthyretin amyloidosis
CA	Cardiac amyloidosis
CMR	Cardiac magnetic resonance
cCT/CCTA	Cardiac computed tomography/Coronary CT angiography
CT	Computed tomography
DD	Differential diagnosis
ECV	Extracellular volume
EF	Ejection fraction
EMF	Endomyocardial fibrosis
FDG-PET	Fluorodeoxyglucose positron emission tomography
GLS	Global longitudinal strain
HCM	Hypertrophic cardiomyopathy
HFpEF	Heart failure with preserved ejection fraction
LGE	Late gadolinium enhancement
LV	Left ventricle/left-ventricular
LVH	Left-ventricular hypertrophy
LVNC	Left-ventricular non-compaction
LVT	Left-ventricular thrombus
MVO	Microvascular obstruction
NC/C ratio	Non-compacted to compacted myocardial thickness ratio
PET/CT	Positron emission tomography–computed tomography
RELAPS	Relative apical sparing
SSFP	Steady-state free precession
STE	Speckle-tracking echocardiography
T1/T2 mapping	Parametric mapping magnetic resonance sequences
TEE	Transesophageal echocardiography
TTE	Transthoracic echocardiography
UEA	Ultrasound-enhancing agents
wtATTR	Wild-type transthyretin amyloidosis
